# Clinical performance evaluation of SARS-CoV-2 rapid antigen testing in point of care usage in comparison to RT-qPCR

**DOI:** 10.1016/j.ebiom.2021.103455

**Published:** 2021-06-26

**Authors:** Isabell Wagenhäuser, Kerstin Knies, Vera Rauschenberger, Michael Eisenmann, Miriam McDonogh, Nils Petri, Oliver Andres, Sven Flemming, Micha Gawlik, Michael Papsdorf, Regina Taurines, Hartmut Böhm, Johannes Forster, Dirk Weismann, Benedikt Weißbrich, Lars Dölken, Johannes Liese, Oliver Kurzai, Ulrich Vogel, Manuel Krone

**Affiliations:** a Institute for Hygiene and Microbiology, University of Wuerzburg, Josef-Schneider-Str. 2 / E1, Wuerzburg 97080, Germany; b Institute for Virology and Immunobiology, University of Wuerzburg, Wuerzburg, Germany; c Infection Control Unit, University Hospital Wuerzburg, Wuerzburg, Germany; d Department of Orthopaedic Trauma, Hand, Plastic and Reconstructive Surgery, University Hospital Wuerzburg, Wuerzburg, Germany; e Department of Internal Medicine I, University Hospital Wuerzburg, Wuerzburg, Germany; f Department of Paediatrics, University Hospital Wuerzburg, Wuerzburg, Germany; g Department of General, Visceral, Transplantation, Vascular and Paediatric Surgery, University Hospital Wuerzburg, Wuerzburg, Germany; h Department of Psychiatry and Psychotherapy, University Hospital Wuerzburg, Wuerzburg, Germany; i Department of Obstetrics and Gynaecology, University Hospital Wuerzburg, Wuerzburg, Germany; j Department of Child and Adolescent Psychiatry, Psychosomatics and Psychotherapy, University Hospital Wuerzburg, Wuerzburg, Germany; k Department of Oral and Maxillofacial Surgery, University Hospital Wuerzburg, Wuerzburg, Germany; l Leibniz Institute for Natural Product Research and Infection Biology – Hans-Knoell-Institute, Jena, Germany

**Keywords:** SARS-CoV-2, Antigen rapid diagnostic test, PCR, Clinical evaluation, Performance evaluation, COVID-19

## Abstract

*Background:* Antigen rapid diagnostic tests (RDT) for SARS-CoV-2 are fast, broadly available, and inexpensive. Despite this, reliable clinical performance data from large field studies is sparse.

*Methods:* In a prospective performance evaluation study, RDT from three manufacturers (NADAL®, Panbio™, MEDsan®, conducted on different samples) were compared to quantitative reverse transcription polymerase chain reaction (RT-qPCR) in 5 068 oropharyngeal swabs for detection of SARS-CoV-2 in a hospital setting. Viral load was derived from standardised RT-qPCR Cycle threshold (C_t_) values. The data collection period ranged from November 12, 2020 to February 28, 2021.

*Findings:* The sensitivity of RDT compared to RT-qPCR was 42·57% (95% CI 33·38%–52·31%). The specificity was 99·68% (95% CI 99·48%–99·80%). Sensitivity declined with decreasing viral load from 100% in samples with a deduced viral load of ≥10^8^ SARS-CoV-2 RNA copies per ml to 8·82% in samples with a viral load lower than 10^4^ SARS-CoV-2 RNA copies per ml. No significant differences in sensitivity or specificity could be observed between samples with and without spike protein variant B.1.1.7. The NPV in the study cohort was 98·84%; the PPV in persons with typical COVID-19 symptoms was 97·37%, and 28·57% in persons without or with atypical symptoms.

*Interpretation:* RDT are a reliable method to diagnose SARS-CoV-2 infection in persons with high viral load. RDT are a valuable addition to RT-qPCR testing, as they reliably detect infectious persons with high viral loads before RT-qPCR results are available.

**Research in context**

**Evidence before this study**

More than 150 Antigen rapid diagnostic tests (RDT) on the market at the end of February 2021 represent a huge expansion of diagnostic possibilities. We searched PubMED for articles using the term (("COVID-19") OR ("COVID") OR ("SARS-CoV-2") OR ("coronavirus")) AND (("antigen detection") OR ("rapid antigen test") OR ("Point-of-care test")), published between January 1, 2020 and February 28, 2021. 139 results were found evaluating the performance of currently available RDT, with heterogeneous results. Sensitivity values of RDT range from 0·0% to 98·3%, specificity from 19·4% to 100·0%. Some of this data differs greatly from manufacturers’ data. However, these previously published performance evaluation studies were conducted under laboratory conditions using frozen swabs, or in small cohorts with middle-aged participants. Comparable RDT performance data from large-scale clinical usage is missing.

**Added value of this study**

Based on previous examinations the real life opportunities and limitations of SARS-CoV-2 RDT as an instrument of hospital infection detection and control are still unclear as well as further study results are limited in transferability to general public. Our findings show that RDT performance in daily clinical routine is reliable in persons with high viral for punctual detection and isolation of infectious persons before RT-qPCR become available. In persons with lower viral load, or in case of asymptomatic patients SARS-CoV-2 detection by RDT was unsuccessful. The general sensitivity of 42·57% is too low to accept the RDT in clinical use as an alternative to RT-qPCR in diagnosis of COVID-19. Calculated specificity was 99.68%. The results are based on a huge study cohort with more than 5 000 participants including a representative age structure with paediatric patients up to geriatric individuals, which portrays approximately the demographic structure of the local society.

**Implications of all the available evidence**

Due to the low general sensitivity, RDT in clinical use cannot be accepted as an alternative but as an addition to RT-qPCR in SARS-CoV-2 diagnosis. The benefit of early detection of highly infectious persons has to be seen in context of the effort of testing and isolation of false positive tested persons.

Alt-text: Unlabelled box

## 1 Introduction

For more than a year, the COVID-19 pandemic has been a worldwide public health challenge. As well as contact tracing, contact reduction, quarantine, and vaccination, the early testing and detection of infectious persons is key in mitigating the spread of disease [1].

Due to its high sensitivity and specificity, quantitative reverse transcription polymerase chain reaction (RT-qPCR) has served as the reference standard in diagnosing SARS-CoV-2 since the beginning of the pandemic. However, because these tests require a diagnostic laboratory and more than an hour to complete, they are quite costly, and their availability is limited [2].

Antigen rapid diagnostic tests (RDT), technically carried out as lateral flow immunochromatographic assays, have become a widely used alternative to RT-qPCR in SARS-CoV-2 diagnostics [3]. RDT persuade through their point-of-care feasibility, short analysis time, and affordability [4].

This prospective performance evaluation study compares the accuracy of RDT in comparison to RT-qPCR in daily clinical routine, with a main emphasis on sensitivity in highly infectious individuals and specificity in broad screening use.

## 2 Methods

### 2.1 Study setting

The study was performed in a 1 438-bed tertiary care hospital in the district of Lower Franconia, Bavaria, Germany. Data collection period ranged from November 12, 2020 to February 28, 2021.

Data was collected during the second wave of the COVID-19 pandemic in Germany [5]. In the hospital's catchment area of Lower Franconia, the average weekly incidence during the study period was 119.21 per 100 000 inhabitants. The maximum of daily new infections was reported on December 23, 2020. Due to a stricter lockdown, case numbers declined in January 2021 [5], [6].

### 2.2 Test enrolment

RDT and RT-qPCR SARS-CoV-2 testing was carried out from consecutive paired oropharyngeal swabs in the following key situations to prevent SARS-CoV-2 outbreaks in the hospital: Patients were tested on admission to the medical, paediatric, child, and adolescent psychiatric wards, the surgical emergency department, as well as the delivery room independently from COVID-19 typical symptomatology. During the study period, usage of RDT on admission was extended to all other clinical departments of the hospital. Patients and persons accompanying underage patients were tested equally. Employees were tested in case of respiratory symptoms, and after close contacts to SARS-CoV-2 positive persons.

In case of more than one documented RDT per person per day, only the first RDT was included in the study. Patients fulfilling the inclusion criteria on multiple days of the study period due to repeated hospital admissions were tested and included once per visit. RDT on test persons with a recent COVID-19 infection and subsequent deisolation were excluded. This category of persons is likely no longer infectious despite persistent RT-qPCR positivity [7].

### 2.3 Data collection

RDT, RT-qPCR results, and demographic data were documented in the local hospital information system (HIS) SAP ERP 6.0 (SAP, Walldorf, Germany). Persons were categorised by symptoms into patients with typical COVID-19 symptoms according to comparable COVID-19 case definition of the CDC [8] and the ECDC [9] (e.g. fever, dry cough, shortness of breath, new olfactory or taste disorder), and persons without or with atypical symptoms which could be attributed to COVID-19 (e.g. deterioration of general condition, falls, diarrhoea). Secondary infections caused by persons tested false negative by RDT were detected using a search of the hospitals’ infection control database.

### 2.4 Antigen rapid diagnostic tests (RDT)

RDT from three manufacturers were selected by manufacturers’ specifications and availability out of 23 products listed by the German Federal Institute for Drugs and Medical Devices in October 2020:[10]

(I) NADAL® COVID-19 Ag Test (Nal von Minden GmbH, Regensburg, Germany)
(II) Panbio™ COVID-19 Ag Rapid Test (Abbott Laboratories, Abbott Park IL, USA)
(III) MEDsan® SARS-Cov-2 Antigen Rapid Test (MEDsan GmbH, Hamburg, Germany)

All RDT included in the study target the nucleoprotein antigen of SARS-CoV-2 according to the test manuals. Two of the three tests (NADAL® and MEDsan®) were approved for use on oropharyngeal swabs. The Panbio™ RDT is approved for nasopharyngeal swabs only but was used in oropharyngeal swabs in comparison to RT-qPCR for this study. The chosen RDT were distributed to clinical sites depending on availability. All swabs were processed directly at point-of-care without storage according to manufacturers’ instructions by trained medical staff. All operators were trained in oropharyngeal swabbing and RDT procedure by VR, ME, OA, DW, SF, MG, MP, RT, HB, or MK or indirectly by another trained operator.

In case of an invalid result, patients were isolated until a RT-qPCR result became available.

### 2.5 Quantitative reverse transcription polymerase chain reaction (RT-qPCR)

Primary RT-qPCR was carried out in the hospital's virological diagnostic laboratory using different RT-qPCR methods, performed according to the manufacturers’ instructions.

As Cycle threshold (C_t_) values for samples with the same viral load differ strongly between different RT-qPCR methods, all but two RT-qPCR positive samples were retested on the same RT-qPCR system (MagNA Pure 96 (nucleic acid purification), 7500 Real-Time PCR System using FTD SARS-CoV-2 Assay) to ascertain comparable C_t_ values. In two samples tested positive on NeuMoDx™ with high C_t_ values (34·3 and 37·2), not enough material was available for retesting, so they were excluded from viral load analysis.

Two standard S_1_ and S_2_ with a known viral loads of 10^6^ (*ViralLoad(S_1_))* and 10^7^ SARS-CoV-2 RNA copies per ml (*ViralLoad(S_2_))* were tested three times on this reference system and resulted in average C_t_ values of 21·3 (*C_t_(S_1_)*) and 18·2 (*C_t_(S_2_)*). The following formula was used to calculate the viral load of a sample *ViralLoad(Sample)* from its C_t_ value *C_t_(Sample)* and the viral loads *(ViralLoad(S1), Viral Load(S2)*) and C_t_ values (*C_t_(S_1_), C_t_(S_2_)*) of the two standards:

$$ViralLoad\;\left(Sample\right)=ViralLoad\;\left(S_{1}\right)\times {\left(\sqrt[{C_{t}\left(S_{1}\right)-C_{t}\left(S_{2}\right)}]{\frac{ViralLoad\left(S_{2}\right)}{ViralLoad\left(S_{1}\right)}}\right)}^{\left(C_{t}\left(S_{1}\right)-C_{t}\left(Sample\right)\right)}$$

Starting on February 3, 2021, all new RT-qPCR positive samples with sufficient viral load underwent melting curve analysis to detect mutation N501Y, followed by a Δ69-70 deletion PCR to detect variant B.1.1.7. If the mutation N501Y without a Δ69-70 deletion was detected, genome sequencing was performed to detect other variants of concern.

### 2.6 Ethics

The Ethics committee of the University of Wuerzburg waived the need to formally apply for ethical clearance due to the study design as well as the need for informed consent as data was collected as part of the clinical routine (File No. 20210112 01).

### 2.7 Statistics

Data was administered using Excel® 2019 (Microsoft, Redmond WA, USA). All statistical calculations have been performed using GraphPad Prism 9 (GraphPad Software, San Diego CA, USA).

The Wilson/Brown method was used to calculate confidence intervals [11]. For statistical significance evaluation Fisher's exact test and Mann-Whitney U test were used. The two-tailed significance level α was set to 0·05.

### 2.8 Blinding

As RDT positive samples were prioritised for RT-qPCR, results of RDT were known by those interpreting RT-qPCR results in some cases.

### 2.9 Role of the funding source

This study was initiated by the investigators. The sponsoring institutions had no function in study design, data collection, analysis, and interpretation of data as well as in writing of the manuscript. All authors had unlimited access to all data. The first and the corresponding author had final responsibility for the decision to submit for publication.

## 3 Results

### 3.1 Test enrolment

**Fig. 1:**
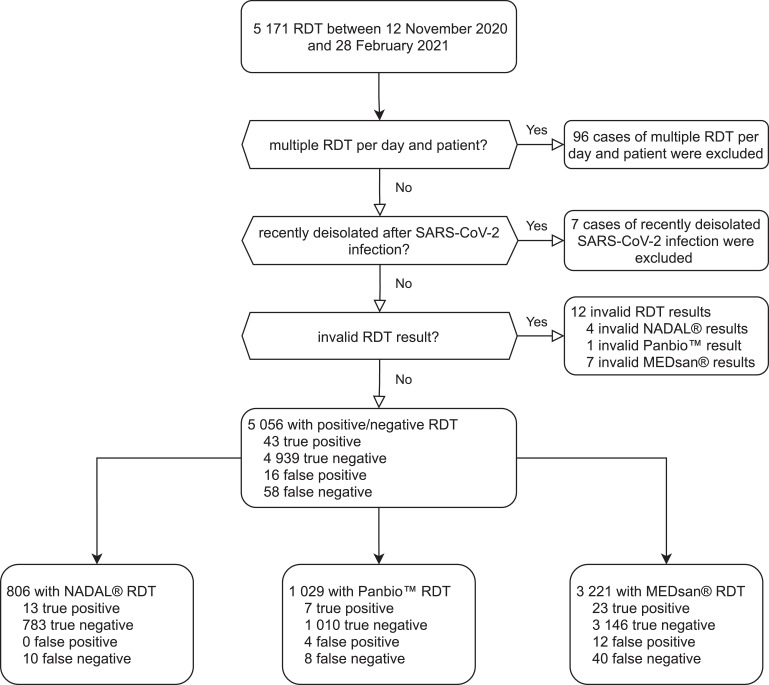
Enrolment of antigen rapid diagnostic test results in the study. RDT: Antigen rapid diagnostic test. RT-qPCR: Quantitative reverse transcription polymerase chain reaction.

Between November 12, 2020 and February 28, 2021, a total of 5 171 parallel RDT and RT-qPCR were carried out. Because only the first RDT for each person each day was included, 96 tests were excluded. Seven tests were excluded because of persistently positive RT-qPCR results. 5 068 RDT carried out on 4 623 individuals were enrolled and included in the study. NADAL® was used in 810 (15·9%), Panbio™ in 1 030 (20·36%) and MEDsan® in 3 228 (63·7%) tests. Twelve RDT samples were excluded from performance analysis because of their invalid RDT result (negative in the positive control, or interfering lines, 4 NADAL®, 1 Panbio™, 7 MEDsan®, Fig. 1).

### 3.2 Study population

The tested persons were between 0 and 100 years old (median age: 43 years, IQR 24–67 year). 2 677 tests (52·82%) were performed on female, 2 390 (47·16%) on male persons. One test was performed on a person assigned to a diverse gender (0·02%). 4 115 tests were performed on patients (81·20%), 615 on accompanying persons (12·13%), and 338 on staff (6·67%).

**Fig. 2:**
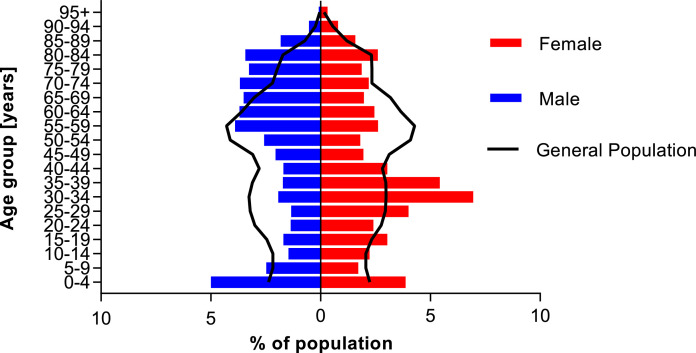
Demographics of the study population compared to the general population of the hospital's catchment area. Study population (red and blue bars, n = 5 067) was compared to the general population of the hospital's catchment area Lower Franconia (black line, n = 1 317 619) as of December 31, 2019. Due to privacy reasons, one person with diverse gender was excluded from the figure. No data on population with diverse gender was available for Lower Franconia. Population data were obtained from Bavarian federal office for statistics [6]

Fig. 2 compares the demographics of the study population to the general population. 22·10% of all tested persons were younger than 20 years, 9·41% were 80 years, or older.

### 3.3 Performance of RDT in comparison to RT-qPCR

Out of 5 056 analysed RDT/RT-qPCR pairs, 101 samples tested positive by RT-qPCR, resulting in a prevalence of 2·00%. 59 (1·17%) samples tested positive by RDT. Thus, 43 samples (0·85%) were assessed true positive, 4 939 true negative (97·69%), 16 false positive (0·32%), and 58 false negative (1·15%). Three of the twelve RDT samples with invalid results were RT-qPCR positive.

The overall sensitivity of RDT was 42·57% (95% CI 33·38%–52·31%), the specificity 99·68% (95% CI 99·48%–99·80%). The positive predictive value (PPV) was 72·88% (95% CI 60·40%–82·56%), and the negative predictive value (NPV) 98·84% (95% CI 98·50%–99·10%).

### 3.4 Comparison of manufacturers

**Fig. 3:**
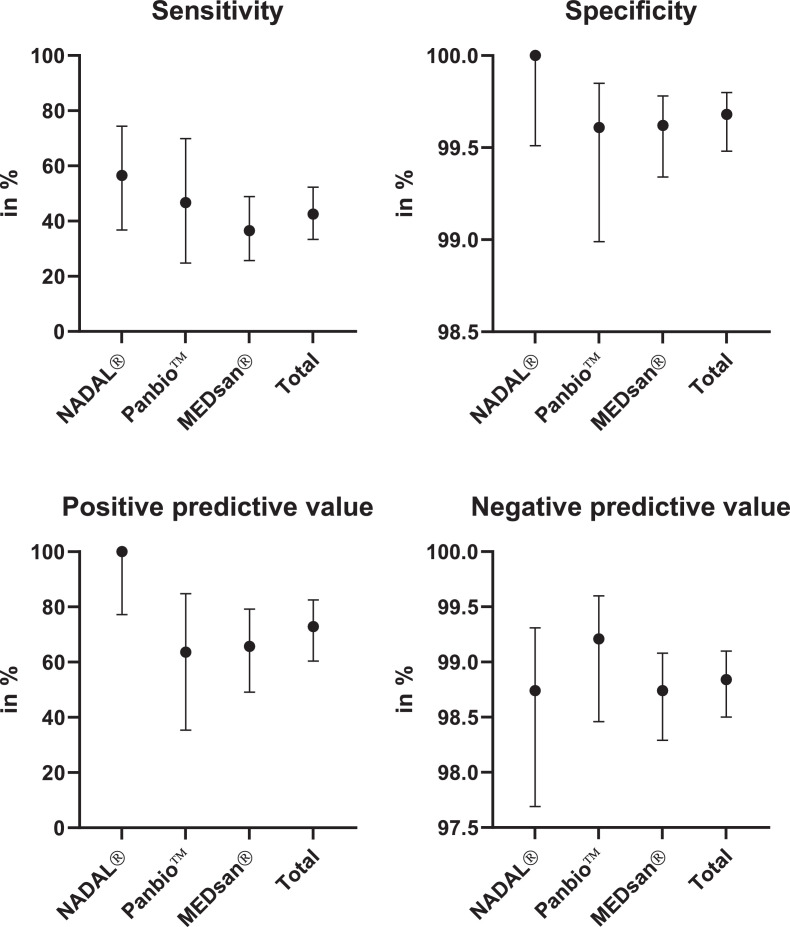
Antigen rapid diagnostic test performance compared to quantitative reverse transcription polymerase chain reaction by manufacturer. Sensitivity, specificity, positive predictive value and negative predictive value of antigen rapid diagnostic tests from three manufacturers (nal von minden NADAL®, Abbott Panbio™, MEDsan®) in comparison to quantitative reverse transcription polymerase chain reaction, n = 5 056. RDT from the different manufacturers were conducted on different samples (806 NADAL®, 1 029 Panbio™, 3 221 MEDsan®). As performed on different samples, test performance can only be compared indirectly.

Sensitivity ranged from 36·51% (23/63, 95% CI 25·72%–48·18%) for MEDsan® over 46·67% (7/15, 95% CI 24·81% to 69·88%) for Panbio™ to 56·52% (13/23, 95% CI 36·81%–74·37%) for NADAL®. Specificity ranged from 99·61% (1 010/1 014, 95% CI 98·99%–99·85%) for Panbio™ over 99·62% (3 146/3 158, 95% CI 99·34%–99·78%) for MEDsan® to 100·00% (783/783, 95% CI 99·51%–100·00%) for NADAL® (Fig. 3).

### 3.5 Relation to viral load

**Fig. 4:**
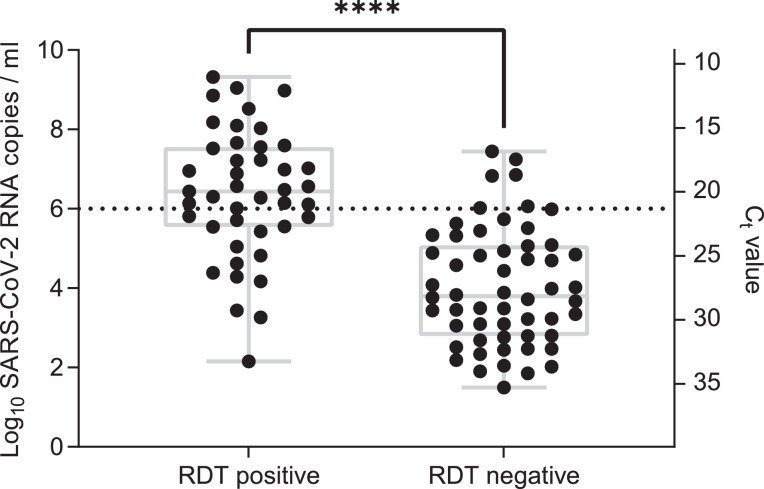
Antigen rapid diagnostic test result in comparison to viral load. Viral load was determined by quantitative reverse transcription polymerase chain reaction (RT-qPCR, n=99), dotted line: viral load of 10^6^ SARS-CoV-2 RNA copies per ml. Due to limited sample volume two samples could not be retested using the reference RT-qPCR method. ****: p<0•0001 (Mann-Whitney U test) RDT: Antigen rapid diagnostic test C_t_: Cycle threshold

C_t_ values in 99 samples tested on the reference system ranged from 11·01 to 35·25 (mean 24·22; SD 5·97), calculated viral loads from 3·16×10^1^ to 2·09×10^9^ SARS-CoV-2 RNA copies per ml. Viral loads in RDT positive persons (median viral load, 2·73×10^6^ copies per ml; range, 1·44×10^2^ to 2.09×10^9^) were significantly higher compared to RDT negative persons (median viral load, 6·23×10^3^ copies per ml; range, 3·16×10^1^ to 2·77×10^7^, p<0·0001 (Mann-Whitney U test), Fig. 4).

**Fig. 5:**
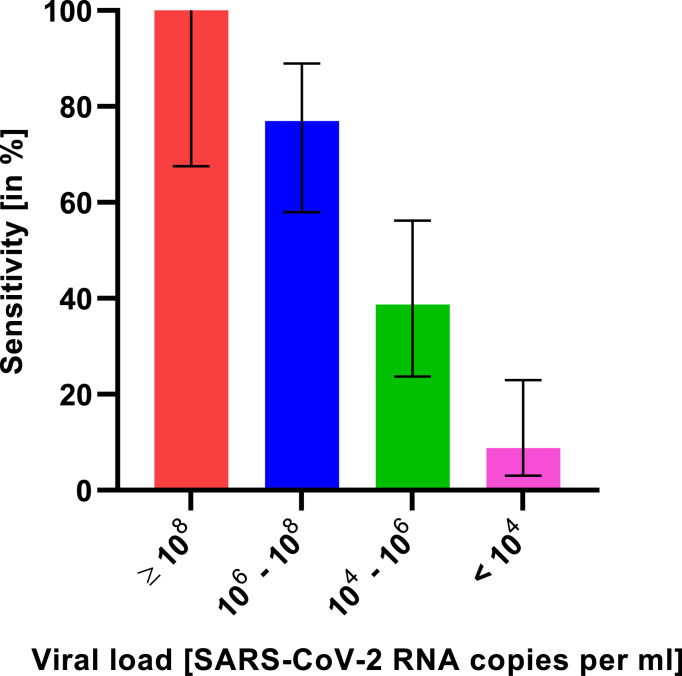
Sensitivity of antigen rapid diagnostic testing in relation to viral load. Viral load was determined by quantitative reverse transcription polymerase chain reaction (n=99)

Sensitivity was 100% in samples with a viral load of ≥10^8^ SARS-CoV-2 RNA copies per ml (8/8, 95% CI 67·56%–100.00%), 76·92% in samples with a viral load: 10^6^ to 10^8^ copies per ml (20/26, 95% CI 57·95%–88·97%), 38·71% in samples with a viral load of 10^4^ to 10^6^ copies per ml (12/31, 95% CI 23·73%–56·18%), and 8·82% (3/34, 95% CI 3·05%–22·96%) in samples with a viral load <10^4^ copies per ml (Fig. 5).

### 3.6 Relation to spike protein variant

Twenty-three samples were analysed for a N501Y mutation: ten of these (43·47%) showed a mutation as well as a Δ69-70 deletion compatible with variant B.1.1.7. No other spike protein variants were found. RDT sensitivity (40·00%, 4/10, 95% CI 16·82%–68·73%) did not differ from wild type samples, and samples not analysed for N501Y mutation (p=1·00, Fisher's exact test).

### 3.7 Relation to symptoms

Twenty-five of 101 RT-qPCR positive tests (24·75%) were performed on asymptomatic persons and persons with atypical symptoms which may be attributed to COVID-19, and 76 (75·24%) on persons with typical COVID-19 symptoms. Sensitivity (24·00%, 6/25, 95% CI 11·50%–43·43%), and PPV (28·57%, 6/21, 95% CI 13·81%–49·96%) were significantly lower in asymptomatic and atypically symptomatic persons compared to persons with typical COVID-19 symptoms. These showed a sensitivity of 48·68% (37/76, 95% CI 37·78%–59·71%), and a PPV of 97·37% (37/38, 95% CI 86·51%–99·87%). This is in line with higher viral loads in typically symptomatic persons (median: 2·10×10^5^ SARS-CoV-2 RNA copies per ml) compared to asymptomatic or atypically symptomatic persons (median: 9·63×10^3^ copies per ml, p=0·22, Mann-Whitney U test).

### 3.8 Secondary infections

One secondary infection was detected in a patient who was placed in a two-bed room with an asymptomatic patient after a false negative RDT result (viral load: 6·70×10^6^ SARS-CoV-2 RNA copies per ml).

## 4 Discussion

Our study proves that combining RDT with an RT-qPCR-based test strategy is useful for early detection of persons with high viral load. This allows the quick identification and isolation of highly infectious persons before RT-qPCR results are available.

The overall RDT sensitivity of 42·6% differs dramatically from the manufacturers’ information of all three RDT ranging from 92·5% (MEDsan®, C_t_ value specified) over 93·3% (Panbio™, no C_t_ specified) to 97·6% (NADAL®, C_t_ value 20–30). Specificity for all used RDT was above 99·6%, which is comparable to manufacturers’ data (99·4% (Panbio™), 99·8% (MEDsan®), or >99·9% (NADAL®)). Sensitivity and specificity data is comparable with performance data from other studies: The Panbio™ RDT has been evaluated in several studies, [12], [13], [14], [15], [16], [17], [18], [19], [20], [21] and reported sensitivity values range from 44·6% [12] to 91·7% [13]. The specificity was continuously in the range of 98·9% [13] to 100%. [14], [15], [16], [17] Three small laboratory or cohort studies are published on NADAL®. [18], [19], [20] Overall sensitivity ranged between 24·3% [19] and 73·1% [20], and test specificity estimated at more than 99% [18], [19], [20]. MEDsan® RDT has so far only been assessed in one published analysis, with a sensitivity of 45·8%, and a specificity of 97·0% [12].

Our data confirms that sensitivity of RDT strongly depends on viral load. Although sensitivity is less than 10% in samples with a low viral load, it reaches 100% with a viral load of more than 10^8^ SARS-CoV-2 RNA copies per ml. As the latter defines potential super-spreaders, it is crucial to identify those individuals as quickly as possible to prevent hospital outbreaks [22]. The low sensitivity of RDT in persons with low viral loads means these tests must be combined with RT-qPCR. Persons may have a low viral load, and not be infectious, at the end of a previously undiagnosed COVID-19 infection [7]. In contrast, viral load at the beginning of a SARS-CoV-2 infection is low, and rapidly increases after the test is performed. Unless these individuals are identified by a parallel RT-qPCR, a false negative RDT may cause and fuel outbreaks [23]. Additionally, incorrect swabbing may strongly decrease in-vitro viral load in the sample and falsely suggest a lower viral load according to a preprint analysis [24]. Because they are more susceptible to false negatives with low viral loads, RDT are more prone to sampling problems. For the same viral load, C_t_ values strongly vary between different RT-qPCR systems. To enable comparisons between RDT sensitivity data, future researchers are advised to mind standardised viral load determination instead of only stating C_t_ values.

The SARS-CoV-2 prevalence in the study population of 2 % was higher compared to the general population but lower compared to studies mainly conducted in symptomatic patients [[14], [15], [16], [17],^21^]. PPV was significantly higher in symptomatic persons compared to asymptomatic which reflects mainly a presumably higher prevalence but also a higher sensitivity of the tests due to higher viral loads. As the PPV is highly dependent on prevalence in the tested population, false positive RDT results do not pose a relevant threat in populations with high prevalence. However, broad use of RDT in asymptomatic individuals in a low prevalence setting may result in a large number of false positive results. This has to be considered for the interpretation of RDT results as well as in further studies.

We did not use nasopharyngeal swabs as they (i) were perceived as being more unpleasant compared to oropharyngeal swabs, (ii) have been associated with serious complications,[25] and (iii) do not provide advantage with regard to viral load at sampling site [26]. Though only using oropharyngeal swabs, Panbio™ did not perform worse compared to NADAL® and MEDsan® RDT which approved for both nasopharyngeal and oropharyngeal specimens, while Panbio™ is only approved for nasopharyngeal specimens. Despite only being approved for nasopharyngeal sampling, our data indicates that Panbio™ is comparable to the other two RDT in oropharyngeal specimen sampling, which may be better tolerated by patients.

RT-qPCR was positive in three of twelve persons with a documented invalid RDT result. This suggests that persons with atypical lines and thus invalid RDT results should be treated as RDT positive until RT-qPCR results are available.

No differences were found in RDT performance regarding spike protein variant B.1.1.7. This is significant because the proportion of this variant is dramatically increasing worldwide [27].

Our study has several limitations. For each participant was assessed only by one of the three chosen RDT, and therefore different RDT only compared indirectly. The three RDT methods were not uniformly distributed throughout the different clinical departments. Each of these also has an individual patient structure and the start of participant inclusion varied between departments. Despite this, our data represents in vivo experience with RDTs in a large cohort. As several operators undertook sampling as well as RDT testing, differences in sampling, test execution, and interpretation may influence the results despite standardised training. The low incidence of SARS-CoV-2 in our study setting limits the number of RT-qPCR positive persons in the study but reflects a realistic scenario of present and future RDT use. The performance of RDTs in other spike protein variants cannot be assessed as they were not determined in the study population. Given the targets of the assays, however, spike protein mutations are unlikely to affect RDT-detection.

RDT are a reliable diagnostic tool to quickly detect persons with a high SARS-CoV-2 viral load. Usage of RDT can help to detect and isolate potential super-spreaders before RT-qPCR results are available, especially for persons entering the hospital. RDT can also help to accelerate treatment of critically ill patients by ruling out high infectiousness. However, all used RDT were unsuccessful in detecting persons with lower viral load. This problem may be aggravated by inadequate sampling, and can result in failure to detect patients in an early stage of infection (i.e. with low but rapidly increasing viral loads). Thus, sensitivity of RDT is too low to accept its clinical use as an alternative to RT-qPCR in diagnosing COVID-19 when RT-qPCR is available. As also recommended by the International Federation for Clinical Chemistry and Laboratory Medicine the clinical indication, the target population, and the performance of the used RDT product in the individual setting has to considered before planning RDT use. Staff training as well as ongoing internal and external performance evaluation are important tools to improve result quality [28]. In a low incidence scenario, the benefit of detecting highly infectious persons by RDT has to be weighed against the effort of testing and isolating falsely positive tested persons taking into account the SARS-CoV-2 prevalence in the population.

## 5 Funding

German Federal Ministry for Education and Science (BMBF), Free State of Bavaria

## 6 Data Sharing Statement

Individual participant data that underlie the results reported in this article, after de-identification (text, tables, figures, and appendices) as well as the study protocol, statistical analysis plan, and analytic code is made available to researchers who provide a methodologically sound proposal to achieve aims in the approved proposal on request to the corresponding author.

## Declaration of Competing Interest

None of the authors has any conflict of interest.
